# Slum Sanitation and the Social Determinants of Women's Health in Nairobi, Kenya

**DOI:** 10.1155/2015/209505

**Published:** 2015-04-28

**Authors:** Jason Corburn, Chantal Hildebrand

**Affiliations:** ^1^School of Public Health and Department of City and Regional Planning, University of California, Berkeley, Berkeley, CA 94720, USA; ^2^School of Public Health, University of California, Berkeley, Berkeley, CA 94720, USA

## Abstract

Inadequate urban sanitation disproportionately impacts the social determinants of women's health in informal settlements or slums. The impacts on women's health include infectious and chronic illnesses, violence, food contamination and malnutrition, economic and educational attainment, and indignity. We used household survey data to report on self-rated health and sociodemographic, housing, and infrastructure conditions in the Mathare informal settlement in Nairobi, Kenya. We combined quantitative survey and mapping data with qualitative focus group information to better understand the relationships between environmental sanitation and the social determinants of women and girls' health in the Mathare slum. We find that an average of eighty-five households in Mathare share one toilet, only 15% of households have access to a private toilet, and the average distance to a public toilet is over 52 meters. Eighty-three percent of households without a private toilet report poor health. Mathare women report violence (68%), respiratory illness/cough (46%), diabetes (33%), and diarrhea (30%) as the most frequent physical burdens. Inadequate, unsafe, and unhygienic sanitation results in multiple and overlapping health, economic, and social impacts that disproportionately impact women and girls living in urban informal settlements.

## 1. Introduction

Inadequate urban sanitation disproportionately impacts women's health, dignity, and human rights. Millions of urban poor women lack access to adequate water and sanitation even though this is considered a basic human right [1]. The health of women often correlates with the health of children and the health of communities more generally, since many women living in urban informal settlements disproportionately support economic and community activities. According to World Health Organization (WHO), improved sanitation is defined as either a flush toilet connected to either a piped sewer system or a septic system, a flush/pour-flush to a pit latrine, a ventilated improved pit (VIP) latrine, a pit latrine with slab, and/or a composting toilet [2]. WHO estimates that approximately 2.6 billion people worldwide continue to live with inadequate sanitation and the environmental health risks are especially severe for the urban poor living in informal or slum conditions [3].

Women living in poor urban communities, particularly informal settlements often referred to as slums, bear the brunt of inadequate sanitation facilities in cities. While we use the term slum in this paper, we acknowledge and want to emphasize that there is no one definition of urban informal settlement and that the term “slum” can inappropriately and incorrectly label a community as dirty and unhealthy. As we will show using data from the Mathare informal settlement in Nairobi, Kenya, environmental conditions can vary greatly in the same slum and researchers need to be attentive to the relative human deprivation within a city when discussing environmental and health hazards. Nonetheless, the United Nations Human Settlements Programme (UN-Habitat) has defined an informal settlement as an area with “inadequate access to safe water, inadequate access to sanitation and other infrastructures, poor structural quality of housing, overcrowding, and insecure residential status” [4]. The UN also defines a slum as a household or group of individuals in an urban area that lack the following:

- Durable housing of a permanent nature that protects against extreme climate conditions.
- Sufficient living space which means not more than three people sharing the same room.
- Easy access to safe water in sufficient amounts at an affordable price.
- Access to adequate sanitation in the form of a private or public toilet shared by a reasonable number of people.
- Security of tenure that prevents forced evictions.

In this paper we focus on informal settlements and health in Nairobi, where over 68% of the city's residents live in slum conditions and have inadequate sanitation facilities [5]. We highlight the combination of environmental, social, and safety issues associated with inadequate sanitation and related human health impacts on women and children in the slums of Nairobi, Kenya. The issue is particularly important in Kenya, since global burden of disease data over the past two decades show that inadequate sanitation is the sixth leading cause of years of life lost for Kenyan women aged 15–49 and the eighth leading cause of years of life lost for Kenyan men [6]. Furthermore, for Kenyan girls below 5, inadequate sanitation rose from the sixth to the fourth leading cause of death between 1990 and 2010. Thus, the importance of sanitation in shaping health outcomes in Kenya has risen over the past two decades. As in other low- and middle-income nations, Kenya's inadequate sanitation has exerted a major influence upon morbidity and mortality rates with particular significance for women and girls.

A review of the sanitation-related health impacts in slums is particularly crucial now that Kenya's 2010 Constitution recognizes that every Kenyan has a right “to accessible and adequate housing, and to reasonable standards of sanitation” [7]. In addition, Kenya's 2007 National Environmental Sanitation and Hygiene Policy stated that, “as a basic human right all Kenyans should enjoy a quality of life with dignity in hygienic and sanitary environments and be free from suffering any ill health caused by poor sanitation” [8]. Yet, from 2003 to 2005, Kenya's sanitation investment was only 2.6% of the total internal investment for water and sanitation allocated in the internal development budget [9]. By 2012, Kenya's sanitation investments ranged from just 0.1% to 0.5% of GDP [10]. Thus, sanitation is usually underfunded and marginalized in programming despite its vital links to improved health, development, and well-being for poor households. According to the Lancet, “sanitation has languished at the bottom of the international agenda [and] the global health community has been complicit in letting it stay there” [11].

## 2. Methodology

This study relied on multiple data sources. First, we reviewed the published literature on slum sanitation and women's health in cities of Sub-Saharan Africa and Kenya specifically. Second, we utilized a household survey of six hundred and fifty residents of the Mathare informal settlement in Nairobi, Kenya, located about 6 kms from the city center. The survey asked questions about household living conditions, expenditures, safety, disease, and self-rated health. Self-rated health was measured by asking the following question: “Compared to other people your age, how would you assess your general health?” The options provided were in ranked order: (1) excellent, (2) very good, (3) good, (4) fair, and (5) poor. In the binary logistic analysis, the categories (1), (2), and (3) were merged as good health, while (4) and (5) were merged as poor health. Therefore the final outcome variable was dichotomous with two levels, namely, “good health” and “poor health.” The data were entered into an electronic database and analyzed using Statistical Package for Social Sciences (SPSS) version 16. Descriptive statistics for categorical and continuous variables were summarized using frequency distributions and percentages for categorical variables, and the mean, standard deviation, mode, and maximum and minimum values for continuous variables.

In addition, the physical locations of environmental sanitation facilities, including water access points, latrines, ablution blocks, pipes, drainage canals, and dumpsites, were mapped across all of Mathare and entered into a Geographic Information System (GIS) database. Spatial analyses included calculating the number of households within a certain distance of sanitation facilities and the mean distance Mathare residents have to travel to reach a latrine. We also report qualitative data from focus group discussions (FGDs) that followed the survey and mapping exercises. We acted as participants and observers while Muungano leaders facilitated over twenty-two community-planning focus groups with Mathare residents. Results include select narratives from these meetings that help make sense of the impacts of inadequate sanitation on the well-being of women and girls in Mathare.

All data were gathered from 2011 to 2013 by community residents that were members of Muungano wa Wanavijiji (Muungano) with the assistance of Muungano Support Trust (MuST), two nongovernmental organizations in Kenya. These organizations partnered with the University of California, Berkeley, and the University of Nairobi to complete the data gathering and analyses. The Institutional Review Board for the Protection of Human Subjects at the University of California, Berkeley, approved the survey and focus group protocols as well as the data sharing processes.

## 3. Results

Over sixty-seven percent of survey respondents in Mathare were women but only forty-five percent of women reported good health compared with sixty-two percent of male respondents (Table 1). Households earning less than 10,000 Kenya Shillings (Ksh) per month reported good health (58%) less frequently than households earning more than 10,000 Ksh per month (87%). Only 8% of Mathare households own their home, but 87% of structure owners reported good health compared to only 19% of renters. While the type of toilet access varied across Mathare, we found that 64% of households using a public flush toilet reported poor health compared to 83% of households relying on pit latrines and 88% of those forced to defecate in the open. Over 83% of households in Mathare report inadequate privacy or no privacy when using a toilet and of these only 7% reported good health.

Having reliable water, defined as twenty-four-hour access seven days a week, resulted in 72% of households reporting good health. Unreliable water access is the reality for over 83% of Mathare households and 88% of these reported poor health. We found that an average of 108 households shared one public water tap in Mathare. We found that 86% of households dispose of wastewater into the street that drains into local rivers. Similarly, 88% of households did not have any organized solid waste collection. Eighty-two percent of households with organized solid waste collection reported good health. Close to three-quarters of Mathare households report not feeling safe in their community and 75% of those that do not feel safe reported having poor health.

**Figure 1:**
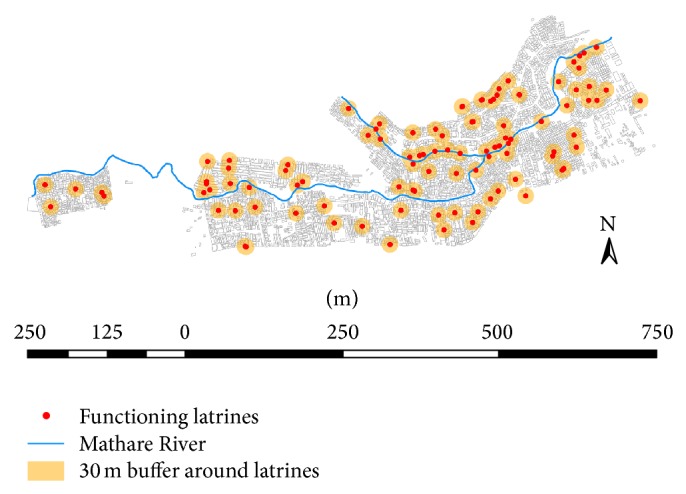
Map of toilets with 30-meter buffer in Mathare Slum, Nairobi, Kenya.

Our mapping data highlights the distribution of toilets across the entire Mathare informal settlement and the area within a 30-meter radius of each toilet facility (Figure 1). We found that an average of eighty-five households shared one toilet and that the mean distance between a household and a functioning toilet was 52 m. Ninety-two percent of households within 30 meters of a toilet reported good health but only 33% of households farther than 30 meters of a toilet reported good health.

We extracted self-reported physical complaints from women respondents (Table 2). The most frequently reported complaint was experience with violence (68%) followed by respiratory illness/cough (46%), diabetes (33%), diarrhea (30%), fever (22%), malaria (23%), typhoid (17%), skin rash (15%), and HIV (14%). Three hundred and twelve (48%) of households reported that a child household member had been ill with one of five diseases (diarrhea, malaria, typhoid, skin infections, or respiratory tract infection) in the past six months.

## 4. The Pathways between Inadequate Slum Sanitation and Women's Health

Our focus group discussions and literature review help explain some of the social conditions that drive the survey responses.

### 4.1. Inadequate Sanitation and Diarrheal Diseases

Our survey found that 30% of women reported at least one episode of diarrheal disease within the previous month. Diarrheal disease was also mentioned as the most frequently reported illness for children. According to WHO, more than 1.4 million children below the age of five worldwide die from preventable diarrheal diseases and it is estimated that 88% of these cases are related to unsafe water or poor sanitation [12]. Fecal contamination in urban slums contributes to high rates of cholera, typhoid fever, dysentery, and intestinal parasites [13]. In urban Kenya, 72% of childhood disease is linked with environmental conditions including poor sanitation and bacteria from informal drainage and fecal contamination of food and drinking water [14]. The prevalence of diarrhea among children below three years of age in Nairobi's slums is 31%, almost double the prevalence present in the rest of Nairobi [14]. Of the 19,500 reported annual deaths in Kenya due to diarrheal disease, 90% were related to poor sanitation and about 65% of those deaths were girls [15]. For instance, the Centre for Microbiology Research in Nairobi found that over 25% of children below 5 years of age in one Nairobi slum had at least one poor sanitation-related intestinal parasite while 12–54% of women living in Nairobi's slums are estimated to have at least one intestinal parasite [16].

### 4.2. Malnutrition and Food Contamination

We heard in focus groups with Mathare residents that inadequate slum sanitation contributes to human waste frequently draining into streets and walkways and is suspected of increasing exposure to food borne pathogens and contributing to childhood diarrhea. One woman noted the following.

The children are often playing in the streets where the waste [human] drains from toilets. There is no sewer here that works. There is no place for hand washing and clean water is another cost. The cost of each toilet use [about 5 Ksh] means our children cannot use them. I have four children and I can't pay for each to use a toilet a few times a day. They come home and touch food, and me, and I worry this is spreading disease.

Children in Nairobi's slums experiencing chronic diarrhea often fail to absorb nutrients from food, contributing to malnutrition and stunting [17]. Sustained or long-term exposure to excreta-related pathogens including helminths or worms in early life limits cognitive or brain development and lowers long-term disease immunity [18]. In 2009, Kenyan children in households with an unimproved toilet were 1.3 times more likely to be stunted than children with an improved toilet [19].

### 4.3. HIV/AIDS and Inadequate Sanitation

Our focus groups suggested that women in Mathare bear a greater burden of managing HIV than men, which has also been documented in other Nairobi slums. In Nairobi, 12% of slum residents are infected with HIV compared to only 5% of nonslum Nairobi residents, but slum women have a 38% higher HIV prevalence than men [20]. Inadequate sanitation and chronic diarrhea can be particularly dangerous for people living with HIV (PLWH) since they are at increased susceptibility to other infectious diseases and opportunistic infections. Slum dwellers living with HIV require an additional 20–80 liters of water daily and frequent access to clean and secure sanitation facilities [21]. WHO estimates that rates of diarrhea are 2–6 times higher among PLWH compared to those who are not infected, and rates of persistent diarrhea are twice as high for PLWH as those in uninfected populations [22]. Furthermore, diarrhea can reduce the absorption of antiretroviral drugs and can speed the progression from HIV to AIDS.

### 4.4. Menstrual Health and Girls' Education

In focus groups with Mathare women and girls, participants mentioned inadequate sanitation during menstrual bleeding as a leading contributor to school absenteeism for girls. In Kenya, less than a quarter of primary and secondary schools met the national standards for the minimal number of latrines per pupil and separate facilities for boys and girls [23, 24]. Inadequate school sanitation in slums can force adolescent girls to miss school to avoid the indignity of public bleeding, finding a private place to change a sanitary napkin, and ridicule by peers when forced to share toilets with boys [25]. One Mathare girl described her situation as follows.

As girls, when we lack enough toilets we have a lot of problems. For example, if our schools do not have toilets where will we dump our pads after using them? This causes girls to stay with one pad for a whole day without changing until she arrives home.

An average of 3.5 million learning days are missed by Kenyan girls per month due to inadequate facilities to manage their menses [26]. Missed school can exacerbate gender inequities and can contribute to greater health vulnerability later in life.

### 4.5. Economic Impacts of Inadequate Sanitation

Inadequate sanitation in Mathare results in economic burdens that include pay-per-use toilets, increased health care/medical costs (i.e., oral rehydration therapy), and decreased wages for women forced to miss work to care for the sick. Most Mathare residents pay 5 Ksh per use, which can present a significant economic burden on slum residents. We found that monthly household toilet expenditures average 305 Ksh, representing about 3% of total household monthly expenditures. However, during an episode of diarrhea, focus group participants reported that increased toilet use combined with treatment expenses and lost wages from missed work can frequently add up to 10% of monthly expenditures. One woman in Mathare noted the following.

My child has it [diarrhea] at least once every two months [which] lasts maybe four or six days. I have to pay for transportation to clinic, medicines, and doctor fees. We need extra fuel to boil more water during these days and I try to get him to use the choo (toilet), but maybe not pay so many times. I usually can't sell my wares at the market on those days, so I loose 40–50* bob* (shillings) maybe.

The World Bank's Water and Sanitation Program found that inadequate sanitation costs Kenya an estimated USD2.7 million annually from lost productivity due to sanitation-related illness and that USD51 million is spent on healthcare related to inadequate sanitation [10].

### 4.6. Indignity and Violence

**Figure 2:**
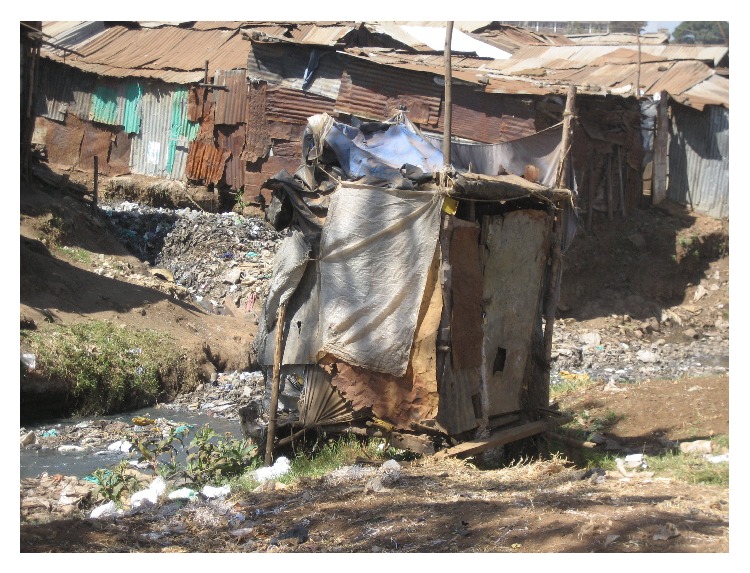
Typical Mathare toilet (source: author photo).

Insecurity and indignity related to inadequate sanitation are key concerns for women and girls in Mathare. Women in our focus groups expressed feeling vulnerable when using public toilets that are far from their homes and that do not have locks on doors or proper lighting at night (Figure 2). Fear of rape, especially at night, can lead to women not drinking fluids, chronic constipation, and using a bucket in their home as a toilet. A young woman in Mathare explained the following.

I always underestimated the threat of violence when regularly using the latrine which all 12 families who live on the plot where I live use. I would go to the latrine at any time provided it was not too late. This was until two months ago when I almost became a victim of rape… You have to walk for about ten minutes to use the latrine… I did not report the incident because one of the four men [who tried to rape me] was well known later told me if I reported the incident to official authorities of the police they would look for and deal with me [27].

According to a study in Nairobi's Kibera slum, over 36% of slum dweller women report being physically forced to have sex (compared to 14% of all Kenyan women) and over 30% of women reported being forced to perform other sexual acts (compared to 14% of all Kenyan women) [28]. A preponderance of evidence suggests that majority of sexual violence in slums occurs in the context of using a toilet, bathing, and/or menstrual hygiene, and in addition to the physical assault, it also leads to increased anxiety, sense of powerlessness and hopelessness, marginalization, and stigmatization. Women in Mathare feel less safe than men, and the ensuing reductions in women's mobility may restrict their personal freedom and access to employment, health, and education and limit their participation in political and recreational activities. Since gender-based violence in Mathare usually goes unpunished, this too can significantly contribute to making and keeping women vulnerable.

## 5. Conclusions

While the links between sanitation and human health are well documented, the disproportionate and overlapping disease, care giving, education, and economic, social, and dignity impacts are rarely captured together for women and girls living in urban informal settlements. Yet, improving slum sanitation can enhance child and maternal well-being, which is particularly important to global urban health since more people are living in cities and women are often primary care givers and household money managers.

We have shown how self-rated health varies by different environmental conditions in Mathare and the spatial distribution of toilet facilities. While additional research is needed to specify the causal links between inadequate sanitation and some of the health outcomes discussed here, we have highlighted that the physical and social environments in urban slums likely interact to coproduce poor health for women. This paper has highlighted the importance of and need for further research to detail the multiple ways women's health can be compromised from a manageable environmental issue, namely, inadequate sanitation. We have also highlighted that the health risks from inadequate sanitation are not unique to the Mathare slum. Environmental engineers and planners in cities of the global south can combine our findings with those from other urban slums to help justify the costs of sanitary improvements that are attentive to the specific needs of women and girls.

## Figures and Tables

**Table 1:** Mathare household characteristics and self-rated health.

Variable	Self-rated health	
	Good health frequency (%)	Poor health frequency (%)
Male	132 (62)	81 (38)
Female	196 (45)	239 (55)
Income		
<10,000 Ksh/mo.	218 (58)	158 (42)
>10,000 Ksh/mo.	195 (71)	80 (29)
Housing		
Renter/tenant	114 (19)	486 (81)
Structure owner	44 (87)	7 (13)
Toilet		
Public flush (drains to street/river)	32 (36)	57 (64)
Pit latrine	85 (17)	302 (83)
Open defecation	21 (12)	151 (88)
Distance from home <30 m	300 (92)	26 (8)
Distance from home >30 m	105 (33)	213 (67)
Privacy (in home)	40 (37)	67 (63)
No privacy	38 (7)	501 (93)
Water		
Reliable yard tap	73 (72)	29 (28)
Nonreliable yard tap	65 (12)	476 (88)
Solid waste		
Organized collection	64 (82)	15 (18)
No organized collection	269 (47)	303 (53)
Security		
Feel safe in community	92 (58)	67 (42)
Do not feel safe	121 (25)	362 (75)

**Table 2:** Physical complaints reported by Mathare women in Nairobi, Kenya (*n* = 435).

Women's physical complaints (*n* = 435)	Frequency (%)
Violence	296 (68)
Respiratory illness (cough)	201 (46)
Diabetes	143 (33)
Diarrhea	131 (30)
Fever	95 (22)
Malaria	101 (23)
Typhoid	74 (17)
Skin rash	65 (15)
HIV	61 (14)
